# Improvement proposals and actions in medication error reports: Quality and strength: A cross‐sectional study

**DOI:** 10.1002/hsr2.70077

**Published:** 2024-09-18

**Authors:** Ville Valkonen, Kaisa Haatainen, Susanna Saano, Miia Tiihonen

**Affiliations:** ^1^ School of Pharmacy, University of Eastern Finland Kuopio Finland; ^2^ Department of Nursing Science University of Eastern Finland Kuopio Finland; ^3^ Hospital Pharmacy, Wellbeing Services County of North Savo Kuopio Finland

**Keywords:** medication errors, medication safety, organizational culture, patient safety, risk management, safety management

## Abstract

**Background and Aims:**

Medication errors (MEs) are a significant source of preventable harm in patient care. Voluntary incident reporting and ME reporting systems are essential for managing medication safety. Analyzing aggregated ME reports instead of individual reports can reveal organizational risks. Organizational culture influences reporting activity and the effectiveness of safety improvements depends on their system‐focus. This study uses aggregated ME reports to investigate the ME management process and reporting culture in medication safety. It aims to create a hierarchy for ME improvement actions and analyze their strength and management flow in aggregated reports.

**Methods:**

A retrospective, cross‐sectional study was conducted to review improvement proposals and actions of ME reports in a Finnish tertiary hospital in 2017–2021. The improvement proposals and actions were categorized into strength classes during three stages: reporter proposals, manager proposals, and documented actions. The report management flow was analyzed. Descriptive statistics were used to describe the characteristics and the chi‐squared test for categorical variables in the statistical analysis.

**Results:**

A new strength classification hierarchy was created with three classes and corresponding numerical values: “strong (3),” “medium (2),” and “weak (1)” Additionally, categories for “no action (0)” and “vague (0)” were included. Out of 5463 ME reports analyzed, improvement proposals and actions were predominantly weak, ranging from 23.4% to 54.2% across different stages of the management process. A significant proportion had no action included (20.5−49.1%) or were vague (4.2−20.6%).

**Conclusion:**

Analyzing the strength of improvement proposals and actions in aggregated ME reports provides new insights into reporting culture and the ME management. The new combined strength classification hierarchy is a suitable tool for this analysis.

## Highlights

- Aggregation of medication error (ME) reports provides new opportunities to learn from the larger context rather than individual cases.
- Analyzing the strength of improvement actions in aggregated ME reports offers new insights into reporting culture as part of the medication safety culture and the ME management process.
- The new combined strength classification hierarchy is an appropriate tool for strength analysis of aggregated ME reports.

## 1 INTRODUCTION

Medication errors (MEs) are the leading cause of preventable harm in patient care posing a threat to patient safety.^1^, ^2^ In addition to the harm caused to patients, MEs also have a significant economic burden, estimated at $42 billion USD globally and £98 million GBP in the UK annually.^3^, ^4^ The threat the MEs pose to healthcare patients is deemed globally essential as the World Health Organization (WHO) declared the third global patient safety challenge *Medication Without Harm* aiming to reduce the global level of severe, avoidable harm related to medications by 50% between 2017 and 2022.^3^

Voluntary incident reporting and ME reporting (MER) systems play crucial roles in the medication safety management and in the overall medication process improvement within the healthcare organizations.^5^, ^6^ The MER systems are generally utilized by reviewing and dealing with individual cases one by one and therefore lessons learnt are limited to individual cases, but the aggregation of ME reports can provide insight into failure modes and risks, which are not observed by analyzing single reports.^7^ The use of aggregated ME data across different units or organizations could be beneficial, but it is currently limited.^5^, ^8^ Also, the sharing of interorganizational data about MEs could be useful for improving patient safety.^9^

Organizational safety culture consists of many different elements of an organizational system and it influences many aspects of safety management.^10^, ^11^ Error reporting possibilities and reporting culture is one key element in safety culture. Organizations with an open and blame‐free approach to errors and error reporting can have higher reporting activities. Even though the existence of easily accessible MER systems promotes error reporting, it is unclear whether they encourage corrective actions and improvement actions.^9^ Having a high reporting activity is not sufficient by itself.^12^ It is important to analyze the reports and take appropriate actions to make improvements based on their findings. If the error reporting does not lead to recommendations or improvement actions, it can be problematic for the safety culture. Managerial commitment to safety is essential to ensure safety culture, and active leadership role is needed for transformational change which is often crucial to improve medication safety systematically.^11^, ^13^, ^14^

Safety improvement actions can be also evaluated how strong and sustainable they are, based on how system‐focused or person‐focused they are and do the actions effect the whole organization, an individual work unit or an individual professional.^15^, ^16^, ^17^, ^18^ This approach has commonly been employed to assess RCA (Root Cause Analyses) strategies instead of ME report improvement actions.^15^, ^16^, ^17^, ^18^ The International Safe Medication Practice (ISMP) also has its own risk reduction strategy hierarchy focusing on medication safety and with many similarities to those employed in RCA.^19^ However, to our knowledge this hierarchy hasn't been systematically used to evaluate aggregated ME reports. There is a need to move towards system‐focused approach from person‐focused approach in healthcare and the aggregation of error reports can guide more system‐focused findings and recommendations.^20^ Organizations may have different approaches to prioritizing safety, but it is important to consider the role of organizational learning in guiding the selection of safety improvement priorities.^21^ Relying solely on externally required activities, such as national requirements, may cause organizations to overlook locally important safety priorities.

The purpose of this study is to utilize aggregated ME reports to investigate ME report management process and to examine error reporting culture as a part of the medication safety culture. The aim of this study is to create strength class hierarchy for ME improvement actions and use it to analyze the quality and strength and the report management process flow of the improvement proposals and actions documented in aggregated ME reports.

## 2 METHODS

### 2.1 Study design and setting

#### 2.1.1 Data collection

This study was conducted as a retrospective cross‐sectional review of ME reports from 2017 to 2021 reported in the voluntary national electronic MER system, HaiPro, at Kuopio University Hospital (KUH). KUH is a medium‐sized tertiary hospital located in Eastern Finland, with approximately 450 beds and over 4000 personnel. The HaiPro system is voluntary and designed for internal safety and quality improvement.^22^ ME reports were recorded into the HaiPro by KUH personnel and all personnel have access to report patient safety incidents, including MEs. At KUH, designated health care professionals, usually department managers, are responsible for managing ME reports. Reporters are requested to include improvement proposals when filing an error report to the HaiPro system. Additionally, report managers are asked to include action plans and proposals while managing the error report. The HaiPro system asks also to document the implemented improvement actions at the end of the management process. Therefore, improvement proposals and actions are recorded in three stages of the error management process. Documenting improvement actions is not mandatory, but recommended and guided by the process. There is no systematic follow‐up of the documentation of improvement actions, except for serious MEs, which have their own systematic process. The study data was exported from the HaiPro system by the researchers. The data included ME reports that were marked as completed.

#### 2.1.2 Data analysis

A new strength classification hierarchy was created because there was no established way of classifying ME report proposals and improvement action. The new strength classification was created by combining three commonly used improvement hierarchies, all of which were also used to present the study results along with the new classification.^16^, ^18^, ^19^ The new combined strength classification hierarchy was formed with three strength classes and corresponding mathematical values: “strong (3)” “medium (2),” “weak (1)” and also categories for no action (0) and vague (0). The classification was included subcategories from the original hierarchies and each improvement proposal and action was classified by using these subcategories.^16^, ^18^, ^19^ The type and strength of improvement proposals in the ME reports were analyzed and coded by the researcher (V. V.), and all ambiguous cases were discussed and resolved by the research team. The analysis and categorization was carried out for each ME report documented in the MER system at all three different stages of the ME report management process: the improvement proposals of the reporter, the improvement proposals of the report managers and the improvement actions documented as having been implemented. If the report contained multiple improvement proposals or actions, the action with the strongest class based on our classification determined the category of the entire ME report.

In this study we also analyzed what happened to the ME reports during the management process by tracking the management process flow of each ME report. The process flow was tracked by observing and analyzing how strong improvement proposals and actions the same ME report had in different management stages.

Descriptive statistics were used to describe the characteristics and the chi‐squared test (*χ* ^2^ test) for categorical variables in the statistical analysis. Results were considered statistically significant when *p* Value was less than or equal to 0.05. The mean value for all improvement proposals and actions in different stages of the management process was calculated using the numerical value defined for the combined strength classes. All data analyses and classifications were conducted by Microsoft Excel 365®.

#### 2.1.3 Ethical considerations

The research permission was granted by the KUH in the autumn of 2021. According to the Finnish National Ethics Committee, ethical approval was not required because the research was based only on anonymous registry data.^23^

## 3 RESULTS

### 3.1 Classification hierarchy

A total of 5 463 ME reports were analyzed and categorized in this study. A combined strength classification was created and used to classify the strength of the ME improvement proposals and actions (Table 1). Table 1 presents the improvement actions included in the combined strength classes used in alphabetical order, along with their original classification hierarchies.

**Table 1:** Classification hierarchy of combined strength class.

Improvement actions	Hettinger^a^	IHI^b^	ISMP^c^	Combined strenght class	Numerical value
Automation, computerization	High	Medium	High Leverage	Strong	3
Barrier	High	Strong	High Leverage	Strong	3
Fail‐safe	High	Strong	High Leverage	Strong	3
Forcing function	High	Strong	High Leverage	Strong	3
Institutional	High	Strong	*NA*	Strong	3
Leadership	High	Strong	*NA*	Strong	3
New devises (usability tested)	High	Strong	High Leverage	Strong	3
Physical environment	Moderate	Strong	*NA*	Strong	3
Audits, review	Moderate	Medium	*NA*	Medium	2
Checklists	Low	Medium	Medium Leverage	Medium	2
Eliminate LASA	*NA*	Medium	*NA*	Medium	2
Increase staffing	*NA*	Medium	*NA*	Medium	2
Redundancies	*NA*	Medium	Medium Leverage	Medium	2
Simplify process	Moderate	Strong	Medium Leverage	Medium	2
Standardization, protocols	Moderate	Strong	Medium Leverage	Medium	2
Warnings, alerts, reminders	Low	Weak	Medium Leverage	Medium	2
“Be more careful”	Minimal	Weak	Low Leverage	Weak	1
Available information	*NA*	Weak	Low Leverage	Weak	1
Disciplinary	Minimal	*NA*	*NA*	Weak	1
Discussion/counseling	Minimal	Weak	Low Leverage	Weak	1
Double check	*NA*	Weak	*NA*	Weak	1
Education using simulations	Low	Medium	*NA*	Weak	1
Educational programs/Training	Low	Weak	Low Leverage	Weak	1
Rules, policies	Low	Weak	Low Leverage	Weak	1
Blank				No action	0
No action				No action	0
Vague				Vague	0

Abbreviations: IHI, Institute for Healthcare Improvement; ISMP, International Safe Medication Practice; LASA, Look alike, sound alike‐drug pairs; NA, not included in the original classification.

^a^ Original classification hierarchy: Hettinger et al.;^16^

^b^ Original classification hierarchy: Institute for Healthcare Improvement IHI. Patient Safety Essentials Toolkit: Action Hierarchy Tool;^18^

^c^ Original classification hierarchy: ISMP: Implement strategies to prevent persistent medication errors and hazards.^19^

#### 3.1.1 Strength of improvement proposals and actions

**Figure 1:**
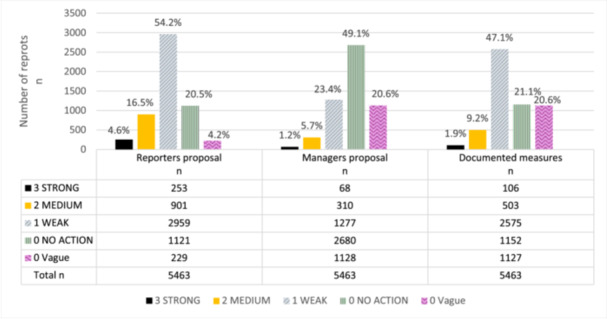
The distribution of improvement proposals and actions strength classification in medication error reports in different management process stages in Kuopio University Hospital in years 2017−2021.

Most of the improvement proposals and actions were weak, vague or suggested no actions (Figure 1).

A major proportion of the proposals and actions were weak actions at all reporting stages, ranging from 23.4 to 54.2%. Furthermore, a high number of improvement proposals were left blank or stating that no action was needed. This was most pronounced for managers' proposals where *No action* class represented almost the half of the reports (49.1%). Respectively, *No action* class accounted for one‐fifth of the reports in the reporter proposals (20.5%) and documented actions (21.1%).

The numerical value of the combined strength class was used to calculate the mean values. The mean value ± standard deviation (SD) for all improvement proposals and actions was 0.70 ± 0.76. The mean value ± SD for reporter proposals was 1.01 ± 0.77, for manager proposals it was 0.38 ± 0.65, and for documented actions, it was 0.71 ± 0.71.

#### 3.1.2 Improvement proposal and action frequency

The improvement proposals and actions were categorized in subcategories of the combined strength class. As shown in Table 2, the most frequent reporter proposals suggested to adhere more strictly to existing rules and policies (*Rules, policies*; 24.3%) and reminded to be more careful (“Be more careful”; 23.0%). Additionally, the reporters expressed quite commonly a desire for standardizations or protocols to harmonize the processes and equipment across the organization (*Standardization, protocols*; 7.8%). Managers often proposed discussing (Discussion/consultation; 12.8%) or reminding people of existing rules and policies (Rules, policies; 4.2%). The most commonly documented improvement actions in the reports were discussing and counseling (27.2%) and reminding employees to follow existing rules and policies (8.5%). Furthermore, organizing education (*Educational programs/Training*; 4.1%) was fairly common action documented in the reports.

**Table 2:** Improvement action and proposals in medication error reports in Kuopio University Hospital in years 2017−2021.

Improvement actions		Reporter proposals		Manager proposals		Documented actions	
		*n*	%	*n*	%	*n*	%
3 Strong	Automation, computerization	115	2.1%	32	0.6%	63	1.2%
	Barrier	1	0.0%	0	0.0%	0	0.0%
	Fail‐safe	2	0.0%	0	0.0%	0	0.0%
	Forcing function	1	0.0%	0	0.0%	1	0.0%
	Institutional	0	0.0%	2	0.0%	1	0.0%
	Leadership	96	1.8%	24	0.4%	22	0.4%
	New devises (usability tested)	22	0.4%	8	0.1%	9	0.2%
	Physical environment	16	0.3%	2	0.0%	10	0.2%
2 Medium	Audits, review	76	1.4%	143	2.6%	218	4.0%
	Checklists	20	0.4%	18	0.3%	33	0.6%
	Eliminate LASA	29	0.5%	11	0.2%	22	0.4%
	Increase staffing	124	2.3%	10	0.2%	16	0.3%
	Redundancies	126	2.3%	16	0.3%	12	0.2%
	Simplify process	14	0.3%	4	0.1%	10	0.2%
	Standardization, protocols	428	7.8%	92	1.7%	173	3.2%
	Warnings, alerts, reminders	84	1.5%	16	0.3%	19	0.3%
1 Weak	“Be more careful”	1258	23.0%	95	1.7%	117	2.1%
	Available information	65	1.2%	80	1.5%	207	3.8%
	Disciplinary	0	0.0%	0	0.0%	0	0.0%
	Discussion/counseling	5	0.1%	700	12.8%	1486	27.2%
	Double check	150	2.7%	82	1.5%	63	1.2%
	Education using simulations	2	0.0%	8	0.1%	15	0.3%
	Educational programs/training	154	2.8%	85	1.6%	224	4.1%
	Rules, policies	1325	24.3%	227	4.2%	463	8.5%
0 No action	Blank	1112	20.4%	2618	47.9%	1109	20.3%
	No action	9	0.2%	62	1.1%	43	0.8%
0 Vague	Vague	229	4.2%	1128	20.6%	1127	20.6%
Total		5463	100.0%	5463	100.0%	5463	100.0%

Abbreviation: LASA, Look alike, sound alike‐drug pairs.

#### 3.1.3 The progress from the reporter improvement proposal to documented improvement actions

Table 3 shows that the majority of the proposals made by the reporters were downgraded from strong to weaker actions during the process. Only 9.9% of the proposals remained classified as strong. Of the proposals initially classified as strong, 38.7% were implemented as weak actions, 20.2% were vaguely documented, and 17.4% had no action documented. Approximately 20% of the reporter proposals, which included any proposed action (classes: 1 Weak, 2 Medium, 3 Strong), did not have any documented actions in the reports at the end of the management process.

**Table 3:** The progress of improvement action strength during the medication error report management process from the reporter improvement proposal to documented actions in Kuopio University Hospital in years 2017−2021.

Documented improvement actions—combined strength class												
	3 Strong		2 Medium		1 Weak		0 No action		Vague/unclear		Total	
	*n*	%	*n*	%	*n*	%	*n*	%	*n*	%	*n*	%
3 Strong	25	9.9	35	13.8	98	38.7	44	17.4	51	20.2	253	100
2 Medium	25	2.8	175	19.4	355	39.4	149	16.5	197	21.9	901	100
1 Weak	35	1.2	189	6.4	1498	50.6	624	21.1	613	20.7	2959	100
0 No action	17	1.5	84	7.5	520	46.4	276	24.6	224	20.0	1121	100
0 Vague	4	1.7	20	8.7	104	45.4	59	25.8	42	18.3	229	100
Total	106	1.9	503	9.2	2575	47.1	1152	21.1	1127	20.6	5463	100

#### 3.1.4 Reporting culture

Depending on the stage of the management process, between 4.2% and 20.6% of the proposals and actions for improvement were classified as vague (Table 2). The proposals in the vague category lacked concrete plans or proposals, and the documented improvement actions did not clearly state what had been done to improve the situation. Instead, many of the statements were defensive, either accusing themselves or others of misconduct or simply making hindsight statements about how someone should have acted. There was no proposal for future improvement.

Example translations of manager proposals and documented actions that were classified as vague:

“Although, the situation in the ward was calm, the workload increased momentarily, with several discharges simultaneously.”

“It is not known who put the medicine cup in the wrong place on the tray, either the one who dispensed the medicine or, for example, the nurse of the evening shift when she administered the evening medicine. Fortunately, the double check works before giving the medicine to the patient and thus the wrong medicines did not go to the patient.”

“In surgery clinics we no longer reconcile the medication lists of the patients who are not undergoing a surgery. Previously we did, but the practice was dropped due to a reduction in nurse staffing for saving money.”

“Patients should have their medication list updated at every visit to the doctor, THIS TIME THAT WAS NOT CONDUCTED.”

**Figure 2:**
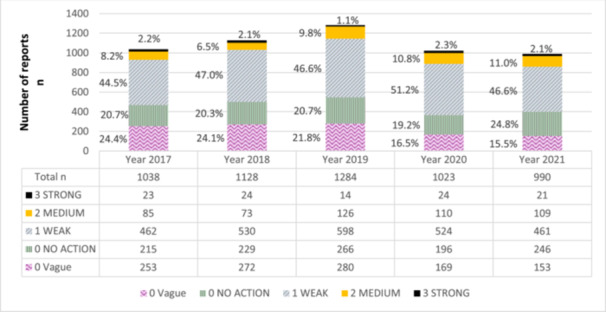
Strength of documented improvement actions of medication error reports in Kuopio University Hospital 2017−2021.

Throughout the study period, the distribution of strength classes for the documented improvement actions remained relatively consistent over the years, as shown in Figure 2.

During the years under examination, there was a slight increase observed in the proportion of improvement actions when focusing on the strongest proposals and actions (classes: 2 medium, 3 strong), increasing from 10.4% to 13.1% (*p* < 0.001, *χ* ^2^ test). Out of the total reports, 91 (1.7%) did not include any improvement proposals from the reporter or manager, nor were there any documented improvement actions.

We compared the strength of the proposals and actions in the near miss events with the errors that reached the patients. The results showed no differences in the types of actions used to manage them or their strength.

## 4 DISCUSSION

### 4.1 Statement of principal findings

To the best of our knowledge, this study is the first to systematically analyzed the strength of improvement proposals and actions of ME reports from an aggregated data set. Previously, this approach has mainly been used to assess methods for enhancing RCA strategies.^15^, ^16^, ^17^, ^18^ ISMP has its own risk reduction strategy hierarchy, which bears numerous similarities to those employed in RCA but focuses on medication safety.^19^ However, as far as we know, it has not been used in the analysis of aggregated ME reports. This study found that conducting a strength analysis of aggregated ME reports is a useful approach to investigate error reporting culture as a part of the medication safety culture and to study error report management processes. Additionally, a new combined strength class categorization hierarchy was developed as part of the study, which proved to be a valuable tool for classifying and analyzing ME reports. The new hierarchy allows the systematic assessment of aggregated ME reports and can be used to investigate the reporting culture and ME report management process. The new combined strength class hierarchy is well‐aligned with the existing ISMP classification and complements it in many ways.

In this study, the majority of improvement proposals and actions were deemed weak, vague, or lacked specific actions to be taken. The mean values for all improvement proposals and actions were clearly below the “weak” classification value. The lowest mean value was for the manager's proposals, while the highest was for the reporter's proposals. The reporters proposed the fewest vague actions, with only a small proportion of proposals lacking clear ideas for improvement. If a reporter had added something to the proposal section, it would usually contain an actual proposal for actions to be taken. In contrast, the manager's proposals and documented actions often lacked detail and were more vague. However, it is important to note that the manager's sections required more concrete actions and proposals with feasible plans, otherwise they were classified as vague. Managers are expected to take action and react to reporters' proposals. The category “Be more careful” received low scores in manager proposals and documented actions. This was due to the fact that if a concrete action was not taken to remind individuals to be more careful, it was classified as vague. For instance, the statement “the operation must be carried out more carefully in the future” lacks specificity as it does not mention the action being referred to.

### 4.2 ME report management process

When comparing the strengths of the proposals at different stages of the management process, it is seen that reporters propose stronger actions (classes: 2 medium and 3 strong) more frequently than managers or the actions that were ultimately documented in the reports. The majority of the strong proposals made by reporters were implemented as weaker actions, had vague statements or no action documented. More than a third of strong reporter proposals were implemented as weak actions. On the other hand, it is worth noticing that more than half of the vague or empty reporter proposals resulted in some actions being implemented. However, it is important to mention that although the percentage is higher for proposals upgrading from no action and vague, the actual number of reports is much lower compared to the opposite direction. The downgrading observed in the strength class categorizations may also be partly explained by limited resources. When planning and implementing actions, it is important to consider the limitations of available resources. While it may be tempting to hope for complex improvements such as setting up a new patient information system for the entire healthcare system to use or doubling staffing, it is important to be realistic about what can be achieved.

A previous study suggests that aggregating patient safety incident error reports can lead to more system‐focused findings and recommendations.^20^ In our study, we discovered that aggregating and analyzing the strength of ME reports can also be used to investigate the safety culture and the ME report management.

When looking at the documented actions by year in this study, there was a slight increase in the proportion of medium and strong improvement actions. Simultaneously, there was a small decrease in the proportion of actions in the no action and vague classes. These findings suggest a positive trend in the improvement management. When compared to the study by Anderson et al, which focused on a regional ME data sharing system in the United States, the figures were more similar. In their study, 45% of all ME reports initially had corrective actions reported.^9^

### 4.3 Reporting culture

Although the national MER reporting system (HaiPro) investigated in this study has been in use in Finland for over 15 years, the study results indicate quite poor learning from the errors. The data indicates challenges in the reporting culture. Many improvement proposals, which were reported appropriately, never turned into actual improvement actions during the ME managing process. The reports in our study data also contain many vague statements documented as proposals or actions indicating that the MER system was viewed as a system for identifying culprits and highlighting errors, rather than as a tool to promote learning and safety development. For instance, some reports featured statements from the manager suggesting that the entire report was unnecessary because no harm had been caused to the patient. Additionally, numerous ME reports with vague proposals and actions only explained how the situation was temporarily resolved, rather than providing actual proposals or actions for improvement. As a result, no ideas for improvement were considered to prevent similar incidents in the future. This type of first‐order problem solving, known as “fixing and forgetting” has also been observed also in other studies and is particularly common with near miss events.^24^ Although this kind of attitude was observed in the study data, there was no difference in the number of strong actions taken to manage the errors that reached patients or the near miss events. However, it is also good to note that original classifications of ME reports have been discovered to be inconsistent in many different MER systems.^5^, ^8^, ^25^, ^26^, ^27^, ^28^ Therefore, there may be variation in how the reports have been classified as near miss events or events that reached the patient.

In healthcare, there is often an emphasis on individual healthcare professionals taking personal responsibility to solve problems as they arise, without considering the impact on the system, which can lead to nonreporting.^29^ These behavioral norms create barriers to organizational learning as it is considered a weakness to seek help or to report problems openly. Efficiency is considered a critical element in healthcare, and staffing resources are often limited. In certain situations, professionals may find it challenging to keep up with their required responsibilities. This can result in a situation where problems are patched up quickly without addressing the underlying issues.^29^ Previous studies have shown that a nonpunitive reporting culture is essential to increasing healthcare professionals reporting activity.^10^, ^30^, ^31^ Other factors that contribute to effective reporting include an easy‐to‐use MER system, adequate education and time resources for reporting, and receiving timely feedback from the reporting.^29^, ^30^

In this study we found that more than half of the ME reports had documented improvement actions. A study by Liukka et al, covering 2011−2015 in Finnish healthcare settings, found that less than 3% of patient safety incidents included a written action to prevent the error from recurring.^12^ They analyzed all patient safety incidents whereas our study focused solely on ME reports. Also, our study evaluated the quality and strength of the proposals while Liukka et al. excluded the weaker actions, such as talking in team meetings and forwarding to another unit or person.

Our study data set also included ME reports that did not contain any suggestions for improvement from the reporter or manager and had no documented improvement actions. These reports raised concerns that some reports may go unnoticed or unreacted. However, the proportion of such reports in the data set was very low.

### 4.4 Strengths and limitations

In our study, we used a comprehensive data set of electronically documented ME reports with multiple information dimensions. This data allowed us to create a detailed overview of the hospital's medication safety and perform various types of analyses. However, the study data contains some limitations that need to be addressed in this research approach. Firstly, it is important to understand the extent of underreporting, as only a limited percentage of ME are even detected and are reported.^32^, ^33^, ^34^, ^35^ Assessing the reported MEs can cause bias as they may not represent completely realistic situations of the units' MEs. It is important to note that our study only analyzed the documented data. It is uncertain how many reports with blank entries have had improvement actions implemented but not recorded in the system. This is because the first priority is to fix the actual problem instead of reporting it. Additionally, it is unclear whether the documented actions have been implemented in practice or if any improvements, such as new policies, have been adopted to prevent the error from recurring. Failing to comprehensively document improvement actions represents a missed opportunity for shared learning. It is important to note that report managers in healthcare settings may not always be experts in patient safety or medication safety, despite being healthcare professionals. In KUH, the reports are primarily managed by chief nurses, pharmacists, and senior physicians in the unit where the ME occurred, with only occasional involvement from patient safety officers or quality managers.

In this study, we have classified only the strongest of the documented proposals or actions to provide a positive assessment of the situation. In many cases, there were also one or several weaker alternatives, or equally viable actions proposed as well. It would be important to consider both long‐term improvement plans for the strongest option and, at the same time, support current practices with lighter solutions that can be adopted. Based on our results, it cannot be directly generalized that the strongest alternative is always the best choice in every situation, as different and multiple actions may be required. However, an assessment can be made regarding the overall safety culture perspective and the degree of system‐focus in the improvement efforts and their practical implementation.

### 4.5 Implications for policy, practice, and research

This study suggests that aggregation and the strength analysis of the ME reports can be used to investigate the safety culture and the management of ME improvement actions. The study also identified challenges in the ME management process. Despite active reporting of MEs, the response to proposed actions was quite weak and person‐centered. However, there was a slight trend towards a more positive direction. It is crucial to ensure that the planned improvement actions will be implemented and that active measurements are in place to track their effectiveness. While the MER system can promote error reporting, it may not necessarily encourage the planning and implementation of improvement actions.^9^ Currently, neither the MER system used in this study nor the management protocol supports the tracking of the progress of the improvement. If the error reporting does result in visible improvements, it can be problematic for the safety culture and undermine reporting motivation.^12^ To ensure healthy safety culture, it is necessary to make improvement actions visible.^12^, ^30^ Active leadership and managerial commitment to safety are needed to make cultural change happen and to establish systematic processes for tracking and measuring the implementation of safety improvement actions.^11^, ^13^, ^14^, ^36^

A successful process from MER to the actual implementation of safety improvement action requires active management and a good medication safety culture. More research is needed to comprehend the barriers and facilitators of the ME management process to enable more effective medication safety improvements. Additionally, it is important to investigate further the possibilities of ME report aggregation and the strength analysis of the ME improvement actions.

### 4.6 Conclusions

Aggregation of ME reports provides not only possibilities to learn across the ME cases from the larger context but also insights to reporting culture as a part of medication safety culture. The new combined strength classification hierarchy enables to investigate the ME report management process by analyzing the strength of the improvement proposals and actions.

## AUTHOR CONTRIBUTIONS

All authors contributed to the study conception and design. Data analysis was performed by Ville Valkonen. The first draft of the manuscript was written by Ville Valkonen and all authors commented on previous versions of the manuscript. All authors have read and approved the final version of the manuscript. Ville Valkonen had full access to all of the data in this study and takes complete responsibility for the integrity of the data and the accuracy of the data analysis.

## CONFLICT OF INTEREST STATEMENT

The authors declare no conflict of interest.

## TRANSPARENCY STATEMENT

The lead author Ville Valkonen affirms that this manuscript is an honest, accurate, and transparent account of the study being reported; that no important aspects of the study have been omitted; and that any discrepancies from the study as planned (and, if relevant, registered) have been explained.

## Data Availability

Due to the nature of data from the confidential error reports, it should not be shared to any third parties.
